# 2D‐Berry‐Curvature‐Driven Large Anomalous Hall Effect in Layered Topological Nodal‐Line MnAlGe

**DOI:** 10.1002/adma.202006301

**Published:** 2021-03-18

**Authors:** Satya N. Guin, Qiunan Xu, Nitesh Kumar, Hsiang‐Hsi Kung, Sydney Dufresne, Congcong Le, Praveen Vir, Matteo Michiardi, Tor Pedersen, Sergey Gorovikov, Sergey Zhdanovich, Kaustuv Manna, Gudrun Auffermann, Walter Schnelle, Johannes Gooth, Chandra Shekhar, Andrea Damascelli, Yan Sun, Claudia Felser

**Affiliations:** ^1^ Department of Solid State Chemistry Max Planck Institute for Chemical Physics of Solids Dresden 01187 Germany; ^2^ Quantum Matter Institute University of British Columbia Vancouver BC V6T 1Z4 Canada; ^3^ Department of Physics and Astronomy University of British Columbia Vancouver BC V6T 1Z1 Canada; ^4^ Canadian Light Source, Inc. 44 Innovation Boulevard Saskatoon SK S7N 2V3 Canada; ^5^ Present address: Department of Physics Indian Institute of Technology Delhi Hauz Khas New Delhi 110016 India

**Keywords:** anomalous Hall effect, Berry curvature, topological nodal‐line MnAlGe

## Abstract

Topological magnets comprising 2D magnetic layers with Curie temperatures (*T*_C_) exceeding room temperature are key for dissipationless quantum transport devices. However, the identification of a material with 2D ferromagnetic planes that exhibits an out‐of‐plane‐magnetization remains a challenge. This study reports a ferromagnetic, topological, nodal‐line, and semimetal MnAlGe composed of square‐net Mn layers that are separated by nonmagnetic Al–Ge spacers. The 2D ferromagnetic Mn layers exhibit an out‐of‐plane magnetization below *T*_C_ ≈ 503 K. Density functional calculations demonstrate that 2D arrays of Mn atoms control the electrical, magnetic, and therefore topological properties in MnAlGe. The unique 2D distribution of the Berry curvature resembles the 2D Fermi surface of the bands that form the topological nodal line near the Fermi energy. A large anomalous Hall conductivity of ≈700 S cm^–1^ is obtained at 2 K and related to this nodal‐line‐induced 2D Berry curvature distribution. The high transition temperature, large anisotropic out‐of‐plane magnetism, and natural heterostructure‐type atomic arrangements consisting of magnetic Mn and nonmagnetic Al/Ge elements render nodal‐line MnAlGe one of the few, unique, and layered topological ferromagnets that have ever been observed.

## Graphical abstract

A layered topological nodal‐line magnet MnAlGe is reported. The material exhibits a large anomalous Hall conductivity driven by the unique 2D Berry curvature distribution. The search for new layered topological magnets and exploration of magnetism and topological properties are enabled by this work.

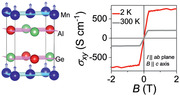

The discovery of materials with outstanding physical properties is important for both fundamental science and the development of next‐generation technological devices. In this regard, topological materials have become the center of focus in solid‐state materials research. The nontrivial topological states arising from the crossing of valence and conduction bands are the origin of numerous exotic transport properties and spectroscopic behaviors.^[^ ^1^, ^2^, ^3^, ^4^, ^5^, ^6^, ^7^, ^8^, ^9^, ^10^, ^11^, ^12^, ^13^ ^]^ Therefore, the realization of new topological materials is crucial for the advancement of topological research and practical applications. Consequently, the recent discovery of magnetic topological materials has created a new frontier.^[^ ^14^, ^15^, ^16^, ^17^, ^18^, ^19^, ^20^, ^21^, ^22^, ^23^, ^24^ ^]^ The interplay between the electronic and spin degrees of freedom can lead to new, correlated topological phases.^[^ ^15^ ^]^ These materials facilitate the observation of anomalous electrical and thermal transport features without external magnetic fields; this renders them ideal candidates for quantized transport, especially when they have high magnetic transition temperatures.

For ferromagnetic (FM) materials, measurement of the Hall effect usually displays an additional component in Hall resistivity when the magnetic field is approximately zero, which is known as the anomalous Hall effect.^[^ ^25^ ^]^ In a topological system, the anomalous Hall effect can be significantly enhanced due to the influence of the Berry curvature of the topological band structure. This has been recently observed in topological magnetic systems including Co_2_MnGa, Co_3_Sn_2_S_2_, Fe_3_Sn_2_, Fe_3_GeTe_2_, Co_2_MnAl, antiferromagnetic Mn_3_Sn, and Mn_3_Ge.^[^ ^14^, ^17^, ^18^, ^26^, ^27^, ^28^, ^29^, ^30^ ^]^ Moreover, layered topological magnets that grow as stacks of 2D magnets, such as Fe_3_GeTe_2_, Co_3_Sn_2_S_2_, and Fe_3_Sn_2_, can be integrated into device applications because of their ease of processability and strong anisotropic magnetism.^[^ ^31^, ^32^, ^33^ ^]^ Therefore, the utilization of 2D‐type or quasi‐2D‐type magnetism in topological systems is interesting. In this study, we used symmetry and crystal‐structure‐based chemical design principles to choose a suitable layered magnet. Heusler compounds provided an ideal starting point because of the ease of tuning their structural and electronic features. Numerous Heusler compounds demonstrate interesting topological band structures containing Weyl crossings, topological insulating states, and other structures.

In **Figure** **1**a, we display the evolution of a layered crystal structure of a 3D Heusler compound (Cu_2_MnAl‐type structure, $Fm\bar{3}m$).^[^ ^34^ ^]^ Tetragonal PbFCl, a ternary ordered variant of the Cu_2_Sb‐type structure (space group *P*4/*nmm*), was obtained by removing every second layer of atoms in the tetrahedral sites of the Cu_2_MnAl‐type structure. Many ternary Mn‐based compounds such as MgMnGe, CaMnSn, SrMnSn, SrMnGe, CaMnGe, and MnAlGe adopt this structure type.^[^ ^35^ ^]^ Except MnAlGe, all of the other mentioned compounds display antiferromagnetic ordering and are semiconductors. MnAlGe is a special example in this class of compounds because it contains an extra valence electron that imparts metallic character and exhibits ferromagnetism at a very high temperature (*T*_C_ ≈ 503 K). In the structure, the Mn atoms (denoted by blue spheres; Figure 1a) formed in 4^4^ square‐net layers that were separated by nonmagnetic Al–Ge spacers. A previous study discovered a topological electronic structure in PbFCl‐type, nonmagnetic ZrSiS and HfSiS with Si‐square nets.^[^ ^36^, ^37^, ^38^ ^]^ The intensively studied superconductor LiFeAs with Fe‐square nets is also an isostructural compound.^[^ ^39^, ^40^, ^41^ ^]^ Herein, we discuss the topological properties of MnAlGe based on first‐principle calculations as well as angle‐resolved photoemission spectroscopy (ARPES) measurements, and their relevance to the electrical and Nernst thermoelectric transport properties. We found that MnAlGe is a topological, nodal‐line, ferromagnet with multiple nodal lines in its electronic structure. The application of spin–orbit coupling resulted in a strong Berry curvature near the Fermi level, leading to a large experimental anomalous Hall conductivity (AHC) of ≈700 S cm^–1^ at *T* = 2 K. Interestingly, we were able to show that the AHC of the individual Mn layers is close to the quantum conductance for a single electron channel of $\frac{e^{2}}{h}$ (3.874 × 10^−5^ S), where *e* is the electronic charge and *h* is the Planck's constant. We ascribe this result to the out‐of‐plane FM order of the layered magnetic structure and the inherent topological nature of the band structure.

**Figure 1:**
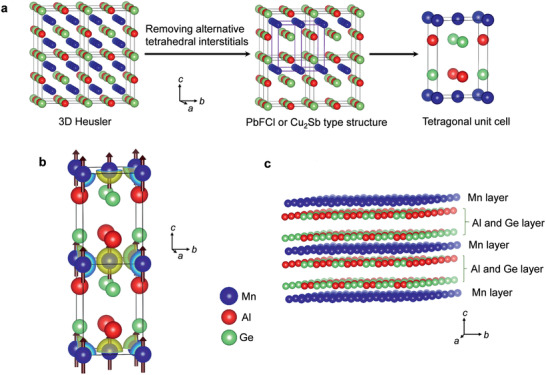
a) Representation of the evolution of 3D Heusler arrangement to a layered structure by removal of alternate tetrahedral interstitials. b) Crystal structure of MnAlGe with an easy axis magnetization direction. MnAlGe crystallizes in a tetragonal structure with space group given by *P*4/*nmm* and lattice parameters *a* = *b* = 3.914 Å, *c* = 5.933 Å. In the structure, Mn atoms occupy special positions 2*a* at (000) and (110), whereas Al and Ge atoms occupy general positions at 2*c* (0 $\frac{1}{2}$ *z*) and ($\frac{1}{2}$ 0 $\bar{z}$), and (0 $\frac{1}{2}$ *z*ʹ) and ($\frac{1}{2}\;\;0\;\;{\bar{z}}^{\prime }$), respectively. The charge density distribution and spin density distribution indicating electronic and magnetic properties are mostly determined by the Mn atoms. c) Representation of layers of Mn (in blue) and Al/Ge (red/green) in the crystal structure. The magnetic Mn and non‐magnetic Al/Ge layers are stacked along the *c*‐axis.

The layered, intermetallic MnAlGe was crystallized in a ternary variant of the Cu_2_Sb‐type structure (i.e., an anti PbFCl structure) (Figure 1b). It displayed a tetragonal structure (space group *P*4/*nmm*; *a* = *b* = 3.914 Å, *c* = 5.933 Å) where the Mn atoms were placed at special positions 2*a* at (000) and ($\frac{1}{2}\;\;\frac{1}{2}$ 0), forming a 4^4^ magnetic square net in the *ab*‐plane. Al and Ge atoms were placed at the general positions 2*c* (0 $\frac{1}{2}$ *z*) and ($\frac{1}{2}$ 0 $\bar{z}$), and (0 $\frac{1}{2}$ *z*′) and ($\frac{1}{2}$ 0 $\bar{z^{\prime }}$), respectively.^[^ ^42^, ^43^, ^44^, ^45^ ^]^ The structure could be viewed as a natural heterostructure consisting of magnetic and nonmagnetic layers stacked along the *c*‐axis (Figure 1c). Specifically, the magnetic Mn atoms aligned within a 2D plane (001) and were separated by two nonmagnetic atomic layers of Al and Ge. The Mn–Mn and Mn–Al/Ge distances in the compound were ≈2.765 and ≈2.778 Å, respectively. The distance between the neighboring Mn planes was equal to the *c*‐parameter, i.e., 5.933 Å.

MnAlGe exhibited a metallic state with both spin‐up and spin‐down states contributing near the Fermi level (**Figure** **2**a). The bands near the Fermi level were dominated by the Mn‐d orbitals for both the spin‐up and spin‐down channels (Figure S1, Supporting Information). Hence, the electrical and magnetic properties of MnAlGe were governed by the square‐net sheets of Mn atoms, while the other two elements (Al and Ge) stabilized the crystal structure. Due to the mirror planes of {M*_x_* |(0.5,0,0)} and {M*_y_* |(0,0.5,0)}, the spin‐down channel formed two nodal lines at band *n* and band *n*+1 (where *n* is the total electron number in the primitive cell) in the *k_x_* = 0 and *k_y_* = 0 planes, respectively, as highlighted in the band structure plot shown in Figure 2b,c. The intrinsic AHC was determined by the integral of the Berry curvature across the entire Brillouin zone (BZ). Therefore, the best way to understand the intrinsic AHE was to consider the Berry curvature distribution in *k*‐space. Considering the magnetic moment along the *c‐*axis and spin orbit coupling (SOC), the double degeneracy of the nodal line lifted and a bandgap opened (Figure S2c, Supporting Information). Meanwhile, a large, 2D‐type cylindrical Berry curvature was generated near this bandgap due to breaking of the band degeneracy by SOC with opening band, anti‐crossing bandgaps (see Figure 2d). Interestingly, the band that constituted the nodal line formed a set of narrow, open, cylindrical Fermi surfaces (FS‐1) (see Figure 2e) extending along the *c*‐axis that exactly matched the Berry curvature distribution. In other words, these 2D Fermi surfaces almost entirely contributed to the AHE with large anisotropy. We theoretically estimated the energy‐dependent AHC of MnAlGe, considering the contribution from the Berry curvature (Figure 2f). A large AHC was observed in the system near the Fermi level. In a MnAlGe with perfect stoichiometry, our ab initio calculation estimates an AHC value of ≈300 S cm^–1^. We further found that electron doping in the system had a large effect on the AHC value and led to a peak value of ≈1000 S cm^–1^, assuming a 60 meV shift of chemical potential. Therefore, the theoretical results indicated that a large AHC can occur in charge neutral or electron‐doped MnAlGe. In addition to the abovementioned, narrow, 2D, Fermi surface responsible for the AHE, two additional sets of Fermi surfaces were observed (FS‐2 and FS‐3) with larger volumes (Figure S3, Supporting Information). Open FS‐3, in particular, covered a significant volume of the BZ and dispersed in all three directions. Due to its large volume, this Fermi surface is expected to dominate the normal electrical transport without significant anisotropy, which will be discussed later here. Since SOC induced a bandgap along the nodal line inside the BZ and a Weyl point was not observed in the system, the band structure in MnAlGe can be viewed as geometrically equivalent to a 3D AHE insulator and we can define an effective, quantized AHC in this bandgap.

**Figure 2:**
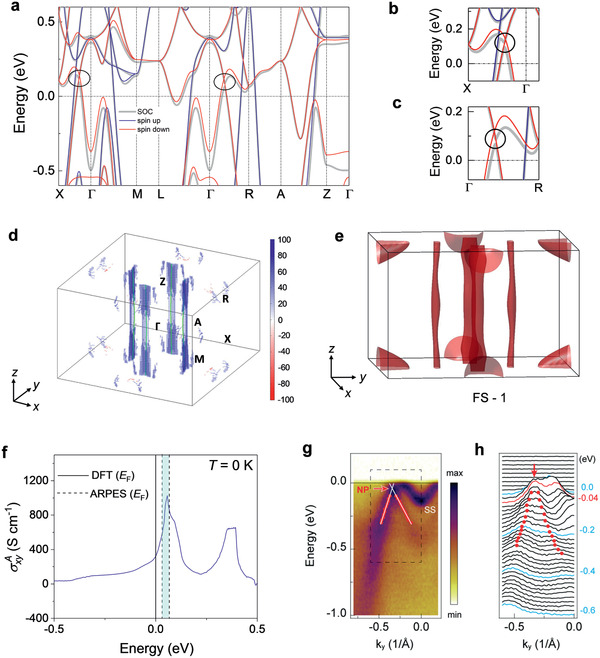
a) Band structure of MnAlGe; the inclusion of spin–orbit coupling results in the loss of the double degeneracy of the bands. Circles highlight the nodal‐line regions; magnified views of these regions are shown in (b) and (c). d) Berry curvature distribution calculated with the inclusion of spin–orbit coupling in the BZ. e) Calculated Fermi surfaces (FS‐1) displayed in the first BZ. f) Theoretically estimated AHC as a function of chemical potential. The dotted lines in the plot indicate the predicted range of AHC based on ARPES measurements. The shaded region is due to the experimental uncertainties in the determination of the NP energy. g) Band dispersion along Γ–*X* measured by ARPES with 60 eV photon at 14 K. The red arrow marks the band crossing NP. The very intense electron pocket centered around the Γ‐point is an SS. See Figure S4, Supporting Information, for details. The momentum distribution curves (MDC) from the dashed rectangular region are presented in (h). The curves are fitted with Lorentz functions to extract the MDC peak positions (red open circles) whose dispersion extrapolates to a crossing point at −46 ± 18 meV.

We have carried out ARPES measurements to investigate the electronic structure of MnAlGe. Figure 2g (also see Figure S5, Supporting Information) represents the band dispersion along the Γ–*X* direction measured by ARPES for surface and projected bulk bands from the [001] surface termination. The ARPES data were taken using 60 eV of circularly polarized light at 14 K. The measured band structure agrees well with the first‐principle calculations with the exception of a slight downshift of the nodal‐line energy. Within the energy resolution of the experiment, we determined the band crossing point to be ≈46 ± 18 meV below the *E*_F_. The intense surface state (SS) that extended to the nodal point (NP) further confirmed the topological origin of the band crossing. The slight discrepancy between the measured and calculated SS dispersion was attributed to the details of surface termination.

MnAlGe is a ferromagnet with a high Curie temperature (*T*_C_ ≈ 503 K) and its ordered structure exhibits strongly anisotropic magnetism along the *c*‐axis (i.e., the easy axis). The observed, ordered magnetic moment is 1.81 μ_B_ per Mn atom.^[^ ^44^ ^]^ The low magnetic moment of the Mn atoms is due to covalent bonding between the Mn and Al/Ge atoms, which facilitates the super‐exchange and the Ruderman–Kittel–Kasuya–Yosida (RKKY) interactions of Mn atoms with the Al/Ge layer. The observation also indicates a strong itinerant nature of d electrons in this ferromagnet.^[^ ^42^, ^43^ ^]^

The temperature‐dependent magnetization measurement with an applied magnetic field of 0.1 T (inset of **Figure** **3**a) demonstrated a paramagnetic to FM transition near 500 K, consistent with previous reports.^[^ ^42^, ^43^, ^44^, ^45^ ^]^ In the magnetic‐field‐dependent magnetization measurement at 2 K, the magnetization saturated rapidly at a value of 2.03 μ_B_ per formula unit at an applied magnetic field of 0.3 T along the tetragonal *c*‐axis, as shown in Figure 3a. When the magnetic field was applied in the *ab*‐plane, saturation of the magnetization required a large magnetic field of ≈ 6 T. Hence, MnAlGe is a highly anisotropic ferromagnet, where the *c*‐axis is the easy axis and the *ab*‐plane is the hard plane for magnetization. Similar behavior was observed at 300 K with a slightly higher saturation magnetization that could be related to the quality of the synthesized crystal.

**Figure 3:**
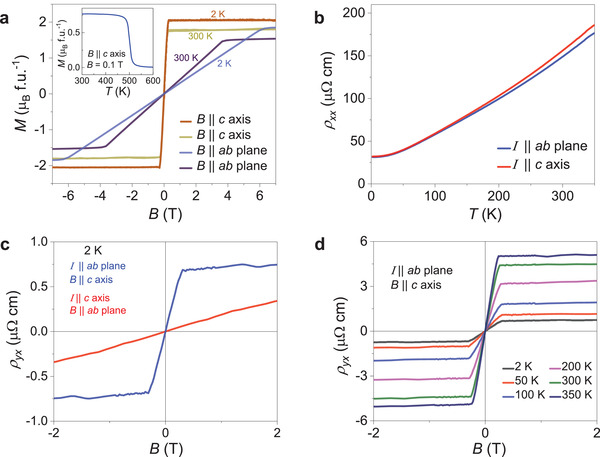
a) Isothermal magnetization curves for MnAlGe with the field along the *c*‐axis and within the *ab*‐plane. The tetragonal *c*‐axis is the easy direction in this compound. The inset shows the temperature‐dependent field cooled magnetization for a field of 0.1 T along the *c‐*axis. The FM transition is observed at *T*_C_ ≈503 K. b) Temperature dependence of the zero‐field resistivity with the current within the *ab*‐plane and *c*‐axis of the crystal. c) Magnetic field dependence of the Hall resistivity (*ρ_yx_*) with the current within the *ab*‐plane and *c*‐axis of the crystal. d) Magnetic field dependence of the *ρ_yx_* at different temperatures. The inset shows the temperature dependence of the zero‐field resistivity with the current within the *ab*‐plane of the crystal.

To understand the effect of the topological states on the physical properties of the sample, we performed electronic transport measurements on the as‐grown MnAlGe crystal. In Figure 3b, we show the zero‐field resistivity (*ρ_xx_*) of the MnAlGe crystal. For both the *I* || *ab‐*plane and *I* || *c‐*axis (inset of Figure 3b) configurations, *ρ_xx_* remained relatively constant. *ρ_xx_* of the sample decreased when the temperature decreased, indicating the metallic nature of the compound. For the *I* || *ab‐*plane at 300 K, a *ρ_xx_* of ≈148 µΩ cm was measured, and reached ≈31 µΩ cm at 2 K, resulting in a residual resistivity ratio (RRR = ρ_300K_/ρ_2K_) of ≈5. The nearly isotropic behavior of the resistivity was due to the dominating influence of the open FS‐3 level that provided the majority of the charge carriers for conduction.

Now, we focus on the Hall effect of MnAlGe. We measured the Hall resistivity in two configurations: 1) magnetic field *B* || *c*‐axis and electrical current (*I*) in the *ab*‐plane; and 2) *B* || *ab*‐plane and *I* in the *c* axis (Figure S10, Supporting Information). The field dependence of the Hall resistivity (*ρ_yx_*) for both of the configurations at 2 K is shown in Figure 3c. The Hall resistivity, when *B* was parallel to the *ab*‐plane, demonstrated linear behavior without any saturation. When *B* was applied along the *c*‐axis, the Hall resistivity increased rapidly in the low magnetic field regime followed by saturation upon further increase of the magnetic field. This result indicated a strong, anisotropic anomalous Hall response in MnAlGe. In the following, we will focus on the transport measurements with *B* || *c*‐axis and *I* in the *ab*‐plane where we observe strong anomalous Hall effect. Figure 3d represents *ρ_yx_* as a function of *B* at various temperatures. The total Hall conductivity (*σ_xy_*) was estimated from the measured Hall resistivity (*ρ_yx_*) and the longitudinal resistivity (*ρ_xx_*) as^[^ ^25^ ^]^

(1) $$\begin{array}{c} \sigma_{xy}\;\;=\;\;\frac{\rho_{yx}}{\rho_{yx}^{2}+\rho_{xx}^{2}} \end{array}$$

The estimated Hall conductivity is presented in **Figure** **4**a (for additional data, see Figures S12 and S13, Supporting Information). In order to estimate the AHC, the high‐field, linear Hall effect was projected towards zero magnetic field. The linear intercept in the Hall conductivity axis is the AHC (Figure S15, Supporting Information). Following a similar process, we estimated the AHC from the *σ_xy_* (*B*) data at various temperatures. A maximum $\sigma_{H}^{A}$ value of ≈700 S cm^–1^ with an anomalous Hall angle (AHA =$\frac{\sigma_{H}^{A}}{\sigma_{xx}}$) of ≈2.18% was measured at 2 K. Furthermore, to understand the large anisotropic out‐of‐plane magnetization and 2D nature of Berry curvature, we have carried out angular‐dependent measurements of the AHE in various current configurations at 2 K (see discussion in Supporting Information and Figure S16, Supporting Information). The observation indicates a nearly constant AHE ($\rho_{yx}^{A},\;\sigma_{xy}^{A}$) at θ ≠ 90° configuration and supports the large anisotropic out‐of‐plane magnetization in MnAlGe.

**Figure 4:**
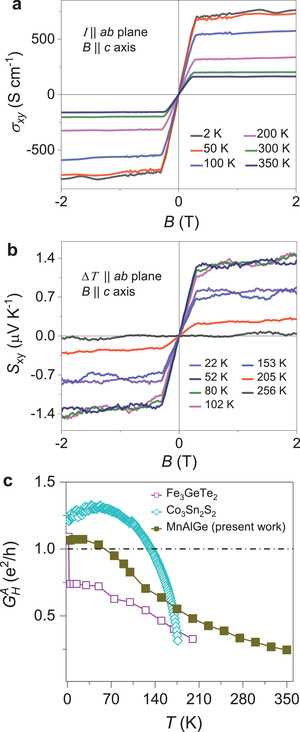
a) Magnetic field dependence of the Hall conductivity (*σ_xy_*). b) Magnetic field dependence of the Nernst thermopower (*S_xy_*) for MnAlGe at selected temperatures. The magnetic field is applied along the *c*‐axis and the temperature gradient along the *ab*‐plane. c) Temperature dependence of AHC per magnetic layer for MnAlGe, Co_3_Sn_2_S_2_, and Fe_3_GeTe_2_.

When a temperature gradient was applied along the sample length, the transverse voltage in the presence of a magnetic field measured the Nernst thermoelectric response. Unlike the Hall conductivity, which accounted for all of the occupied electronic states, Nernst thermopower (*S_xy_*) is sensitive to the electronic states near the Fermi energy. Therefore, it is an important tool for studying topological materials wherein the topological features exist near the Fermi energy, like in the present system of MnAlGe. Figure 4b displays the field‐dependent *S_xy_* of MnAlGe at different temperatures, confirming its anomalous behavior. The temperature gradient was created in the *ab*‐plane and the magnetic field was applied along the *c*‐axis. The shape of the *S_xy_* versus *B* data matches that of the Hall resistivity. We obtained a large, anomalous Nernst thermopower value of *S_xy_* ≈ 1.3 µV K^−1^ at 52 K. The measured Nernst signal was much larger than predicted by the magnetization value (see Supporting Information). This result associates MnAlGe with a number of nontrivial materials that exhibit large anomalous Nernst effects.

We have established that, although MnAlGe is not a van der Waals compound, due to the large separation between the magnetic square net layers of Mn atoms, the AHE is dominated by the 2D electronic features in the band structure. There are three important points we would like to emphasize. First, the electronic states near the Fermi energy were dominated by Mn‐d orbitals, while the s and p orbitals of the nonmagnetic Al–Ge subunit only contributed far from the Fermi energy. The fat bands observed in Figure S2, Supporting Information, clearly show the relative contribution of the orbitals in the band structure. Hence, the transport properties of MnAlGe were entirely determined by the Mn‐square net layers. Second, the unique 2D distribution of the Berry curvature in MnAlGe that arose from the band anti‐crossing of the nodal line perfectly matched the 2D FS obtained from the band constituting the nodal line. Therefore, the AHE observed in this compound originated from the 2D feature of the bands that contained nodal lines. Moreover, the scaling of $\rho_{yx}^{A}$ versus $\rho_{xx}^{2}$ indicates the intrinsic contribution to AHE. It should be noted that the relation of $\rho_{yx}^{A}$ versus $\rho_{xx}^{2}$ is not strictly linear in the temperature dependent AHE data. Therefore, a temperature range 2–100 K has been used for linear fitting to obtain the intercept ≈560 S cm^–1^ as the intrinsic contribution (see discussion in Supporting Information for the departure from linearity, Figure S17, Supporting Information). Based on the ARPES study, the nodal‐line anticrossings was situated just below the Fermi energy that enhanced the AHC. This can be understood from Figure 2f, which shows that shifting the nodal line from above to below the *E*_F_ moves AHC from the base of a peak in the $\sigma_{xy}^{A}$ versus *E* data to almost the peak. This explains the observation of a large AHC in experiments compared to the value predicted by DFT, when the Fermi energy is placed below the nodal line. Finally, the part of the FS that contributed the majority of the charge carriers for conduction exhibited dispersion in all three dimensions. Thus, the electrical resistivity and the ordinary Hall effect will demonstrate a small anisotropy, which is consistent with experimental observations. Next, we discuss how MnAlGe compares with other well‐known 2D, FM, anomalous Hall systems. Quasi‐2D kagome lattice compounds Co_3_Sn_2_S_2_ and Fe_3_Sn_2_ have recently been established as topological Weyl and massive Dirac semimetals. Interestingly, the separation between the Mn layers in MnAlGe (5.93 Å) was larger than the separation between the two Co‐kagome layers in Co_3_Sn_2_S_2_ (4.39 Å) and comparable to that of the Fe‐kagome layers in Fe_3_Sn_2_ (6.60 Å). Notably, while the FS in Fe_3_Sn_2_ is open and quasi‐2D, the FSs in Co_3_Sn_2_S_2_ are 3D. The value of the AHC in MnAlGe was higher than in Fe_3_Sn_2_ (170 S cm^−1^) and comparable to that of Co_3_Sn_2_S_2_ (505–1130 S cm^−1^). In a true, van der Waals, topological nodal‐line, compound Fe_3_GeTe_2_, the separation between the two magnetic layers was significantly larger (8.16 Å), while in Fe_1/4_TaS_2_, which forms by the Fe intercalation of the van der Waals gap of TaS_2_, the separation between the Fe layers (6.07 Å) was approximately equal to MnAlGe. Importantly, the AHC and FM transition temperature of MnAlGe were among the highest reported, as shown in Table S2, Supporting Information.

As the AHE in MnAlGe resulted from the 2D band, we estimated the anomalous Hall conductance per FM Mn layer ($G_{H}^{A}$) by multiplying the bulk AHC by the separation between the two Mn layers. The value of $G_{H}^{A}$ at 2 K was 4.153 × 10^−5^ S, which was close to the quantum conductance for a single electron channel of $\frac{e^{2}}{h}$ (3.874 × 10^−5^ S), as shown in Figure 4c. For comparison, we also estimated this value for the layered compounds Co_3_Sn_2_S_2_ and Fe_3_GeTe_2_. The calculated charge carrier density per sheet of magnetic layer in MnAlGe was ≈ 2.54 × 10^14^ cm^−2^ at 2 K (see Supporting Information) that was one order higher than graphene, which is known to show quantum effects up to room temperature.^[^ ^3^ ^]^ The zero‐field electrical conductivity of MnAlGe in ≈10^4^ S cm^–1^, further specifying that the anomalous Hall transport in this compound, is in the intrinsic regime. The $\sigma_{H}^{A}$ value decreased with increased temperature and reaches ≈190 S cm^–1^ at 300 K (Figure S12b, Supporting Information).

We have demonstrated that FM MnAlGe that is composed of Mn‐square net layers is a topological nodal‐line metal. The electrical transport properties and magnetism in this compound are solely controlled by the Mn layers while the nonmagnetic Al–Ge spacers provide the structural stability. Nodal‐line band anticrossings near the Fermi energy were responsible for a large Berry curvature that was reflected by one of the largest reported AHCs. Importantly, the Berry curvature distribution was uniquely 2D, matching the 2D Fermi surface obtained from the bands constituting nodal lines near the Fermi energy. Hence, the anomalous Hall effect in this compound was mostly 2D. This brings MnAlGe into a class of recently discovered, layered ferromagnets that exhibit large anomalous Hall effects with an additional advantage of a very high FM transition temperature. Our work should motivate the search of new layered topological magnets for anomalous transport properties.

## Experimental Section

### Single‐Crystal Growth of MnAlGe and Characterization

The single crystals of MnAlGe were grown using the Bridgman–Stockbarger crystal growth technique. First, a polycrystalline sample was synthesized by arc melting. As‐purchased high‐quality elemental manganese (99.999%, Alfa Aesar), aluminum (99.999%, Alfa Aesar), and germanium (99.999%, Alfa Aesar) were used for the synthesis. The arc‐melted sample was then crushed into powder and packed in a custom‐designed long alumina crucible with a sharp conical tip that was sealed inside a quartz tube. The tube was heated to 1173 K over 48 h, soaked for 24 h, and then slowly cooled down to 573 K over 7 days. The single crystallinity of the as‐grown crystal was evaluated by white‐beam backscattering Laue X‐ray diffraction (XRD). Sharp spots in the Laue XRD diffraction pattern indicated the single‐crystalline nature of the crystals (Figure S6, Supporting Information). The powder XRD measurement was performed at room temperature using a Huber image plate Guinier G670 camera operated with CuK_α1_ radiation (λ = 1.54056 Å). The powder XRD data on the crushed crystal displayed no unindexed peaks, indicating phase purity (Figure S7, Supporting Information). The composition of the MnAlGe sample was examined using scanning electron microscopy with an energy‐dispersive EDAX analyzer and an ICP‐OES (5100SVDV, Agilent). The stoichiometry of the crystals was close to the nominal composition, as shown in Figure S9 and Table S1, Supporting Information. Well‐characterized and aligned crystals were cut into bar shapes for the electrical transport and magnetization measurements.

### Electrical Transport, Nernst Thermoelectric, and Magnetization Measurements

The electrical transport properties were measured using a PPMS9 instrument (ACT option, Quantum Design). The standard four‐probe method was used for all the measurements. The Nernst thermoelectric measurements were carried out in the one‐heater two‐thermometer configuration. The field sweep measurements were performed in a PPMS instrument with an external nanovoltmeter and current source (Keithley), which were controlled using LabVIEW (National Instruments). In order to correct the contact misalignment, the measured resistivity, Hall resistivity, and Nernst thermopower data were field‐symmetrized and anti‐symmetrized, respectively. The magnetization measurement was performed using a Quantum Design MPMS3 instrument.

### ARPES Measurements

ARPES measurements were performed at the Canadian Light Source on the Quantum Materials Spectroscopy Centre (QMSC) beam line. Samples were cleaved and measured at 14 K in a base pressure of 5 × 10^−11 ^Torr. Photoelectrons were detected by a Scienta R4000 analyzer, with the energy resolution of 30 meV.

### Ab Initio Calculations

The electronic structure calculations were performed based on density functional theory (DFT) using the full‐potential, local‐orbital code with a localized atomic basis and full‐potential treatment.^[^ ^46^ ^]^ The exchange and correlation energies were considered at the generalized gradient approximation level.^[^ ^47^ ^]^ The Bloch wavefunctions were projected into the atomic‐orbital‐like Wannier functions and the tight‐binding model Hamiltonian was constructed. With the tight‐binding model Hamiltonian, the intrinsic AHC was calculated using the linear response Kubo formula approach as follows^[^ ^48^ ^]^

(2) $$\Omega_{n,ij}\left(\vec{k}\right)\;\;=\;\;\text{Im}\sum_{m\neq n}\frac{n\left(\vec{k}\right)|{\hat{\upsilon }}_{i}|m\left(\vec{k}\right)m\left(\vec{k}\right)|{\hat{\upsilon }}_{j}|n\left(\vec{k}\right)-\left(i↔j\right)}{{\left(\varepsilon_{n}-\varepsilon_{m}\right)}^{2}}$$

where $\langle m\left(\vec{k}\right)|$ and $\langle n\left(\vec{k}\right)|$ are the eigenstates, ε is the eigenenergies of the Hamiltonian *H*, and ${\hat{\upsilon }}_{i}$ is the velocity operator.

Subsequently, we calculated the AHC, given by

(3) $$\sigma_{H}^{A}\;\;=\;\;-\frac{e^{2}}{\hbar }\sum_{n}\int \frac{dk}{{\left(2\pi \right)}^{3}}\;\;\Omega_{n,xy}\left(k\right)f_{nk}$$

where *f_nk_* is the Fermi–Dirac distribution for band *n* at point *k*.

## Conflict of Interest

The authors declare no conflict of interest.

## Supporting information

Supporting Information

## Data Availability

Research data are not shared.
