# Supplementary material for: 2D‐Berry‐Curvature‐Driven Large Anomalous Hall Effect in Layered Topological Nodal‐Line MnAlGe
Source: Adv Mater. 2021 Mar 18;33(21):2006301. doi: 10.1002/adma.202006301 (PMC11469258; doi:10.1002/adma.202006301)
Supplement: Supplementary file 1 — Supporting Information [file ADMA-33-2006301-s001.pdf]

© 2021 Wiley-VCH GmbH

ADVANCED MATERIALS

Supporting Information

for *Adv. Mater.,* DOI: 10.1002/adma.202006301

2D-Berry-Curvature-Driven Large Anomalous Hall Effect in Layered Topological Nodal-Line MnAlGe

Satya N. Guin, Qiunan Xu, Nitesh Kumar, Hsiang-Hsi Kung, Sydney Dufresne, Congcong Le, Praveen Vir, Matteo Michiardi, Tor Pedersen, Sergey Gorovikov, Sergey Zhdanovich, Kaustuv Manna, Gudrun Auffermann, Walter Schnelle, Johannes Gooth, Chandra Shekhar, Andrea Damascelli, Yan Sun, and Claudia Felser**

Supporting Information

2D-Berry-Curvature-Driven Large Anomalous Hall Effect in Layered Topological Nodal-Line MnAlGe

Satya N. Guin, Qiunan Xu, Nitesh Kumar, Hsiang-Hsi Kung, Sydney Dufresne, Congcong Le, Praveen Vir, Matteo Michiardi, Tor Pedersen, Sergey Gorovikov, Sergey Zhdanovich, Kaustuv Manna, Gudrun Auffermann, Walter Schnelle, Johannes Gooth, Chandra Shekhar, Andrea Damascelli, Yan Sun, and Claudia Felser**

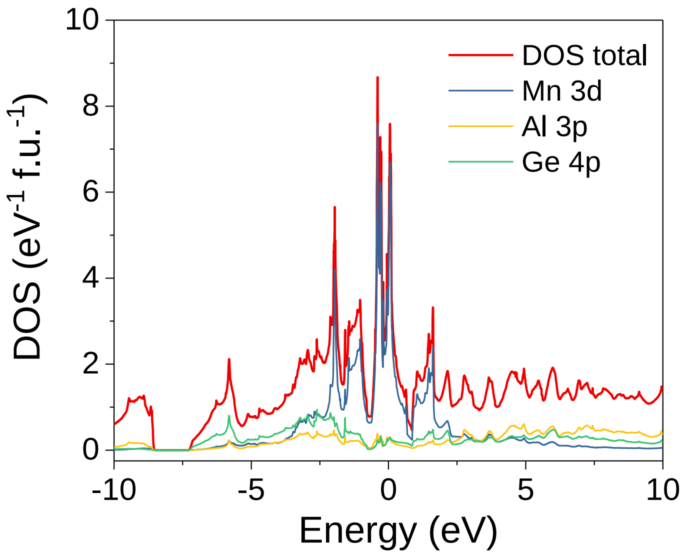

Figure S1. The partial DOS indicates that the bands near the Fermi level are dominated by the Mn-*d* orbital.

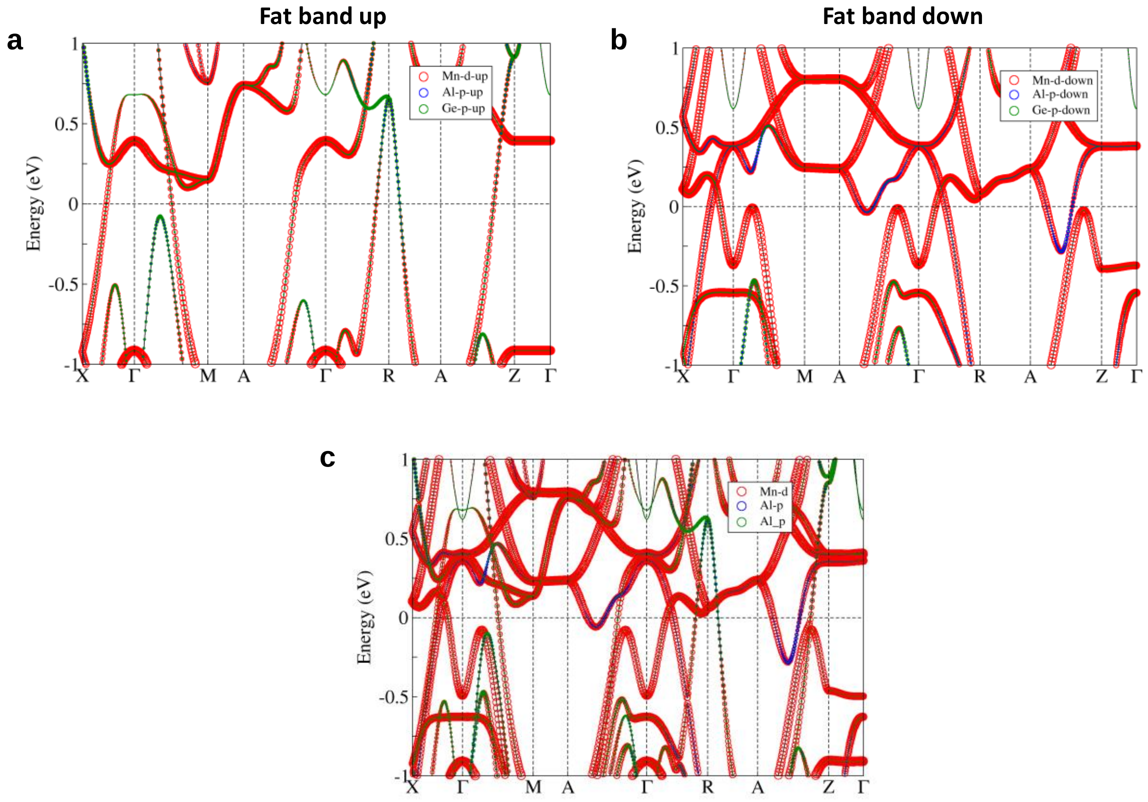

Figure S2. Atomic orbital projected electronic band structure of MnAlGe. (a) spin-up, (b) spin-down channels, and (c) including SOC. The size of the circles in the dispersions represent the contribution weights from the different orbitals. The bands around the Fermi level are dominated by the Mn-*d* orbital.

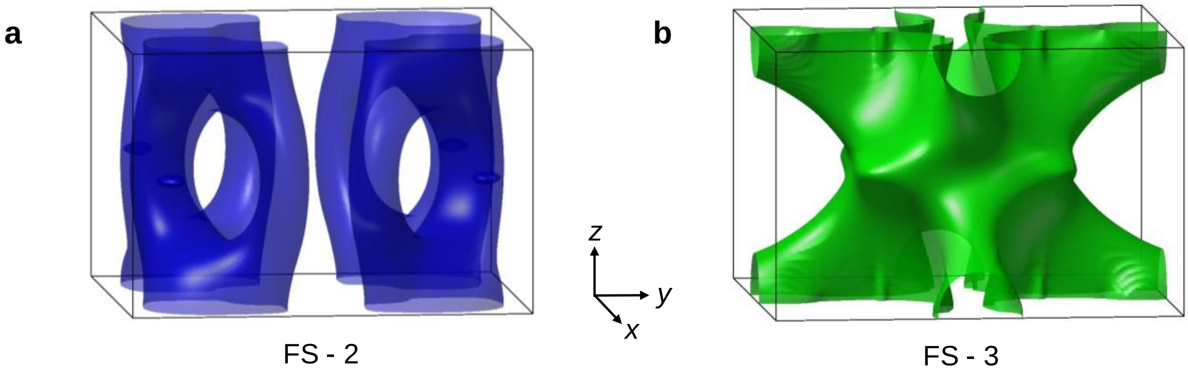

Figure S3. Additional Fermi surfaces (FS) of MnAlGe. (a) FS-2 is an open cylindrical type extending along the *c*-axis. (b) FS-3 covers a significant volume of the Brillouin zone and disperses in all three directions. This open FS dominates in the normal electrical transport without much anisotropy.

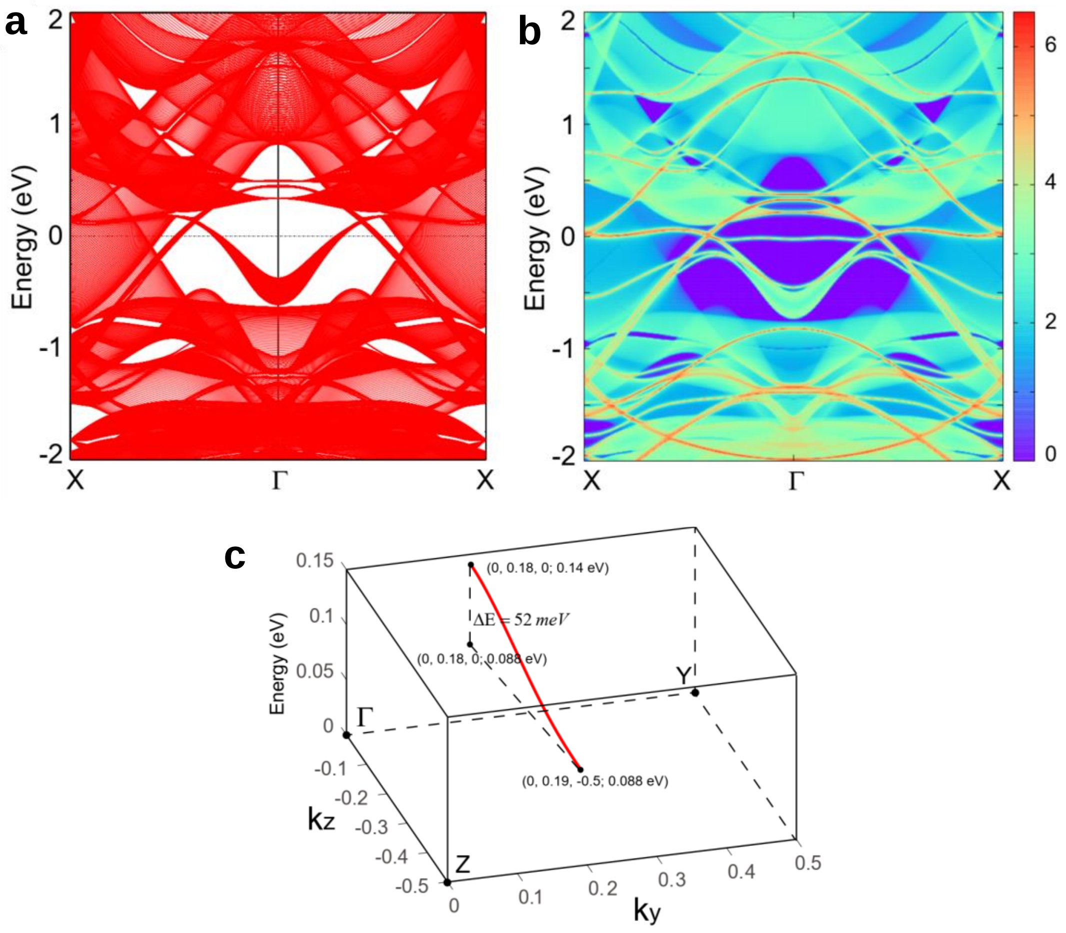

Figure S4. Energy dispersion of MnAlGe. (a) Bulk band structure along high symmetry lines *X*-*Γ*-*X* with k_z-projection. (b) Surface states along high symmetry lines of *X*-*Γ*-*X*. (c) Energy dispersion of nodal line in k_z-direction.

To identify the (001) surface state in MnAlGe, we made a comparison between k_z-projected band structure and half-infinite band structure along the high symmetry lines of *X*-*Γ*-*X*, see Figure S4a,b. From the difference of these two-energy dispersions, one can easily recognize the surface bands around *Γ* point near Fermi level. We also calculated the dispersion of mirror symmetry projected nodal line along *kz*-direction. From Figure S4c we can see that the nodal line is in the constrained in the range of 0.088 eV to 0.14 eV, only 52 meV in the energy space. Hence, this nodal line obviously shows the 2D nature of the electronic band structure.

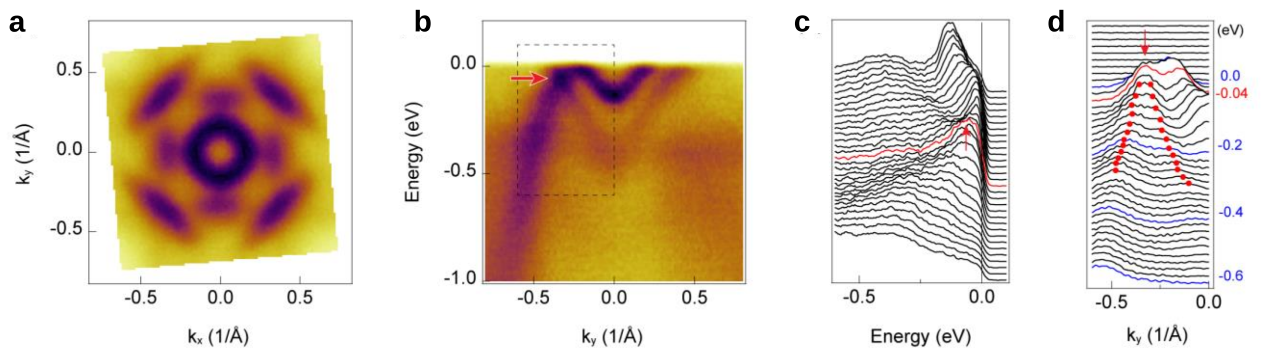

Figure S5. (a) The Fermi surface mapping measured with 60 eV circularly polarized light at 14 K. The data is averaged over 4-fold rotation symmetry. (b) Band dispersion along $\Gamma - X$ (without symmetrization). The red arrow marks the band crossing nodal point. The very intense band around the Γ point is a surface state (see also Figure S4). (c) Energy distribution curves (EDC) and (d) Momentum distribution curves (MDC) from panel (b) in the rectangular box. Fit to the EDC through the nodal point (red) determine the bands crossing point to be about 50 meV below E_F. The MDC curves are fitted with Lorentz functions to extract the peak positions (red open circles), whose dispersion extrapolates to a crossing point at -46±18 meV.

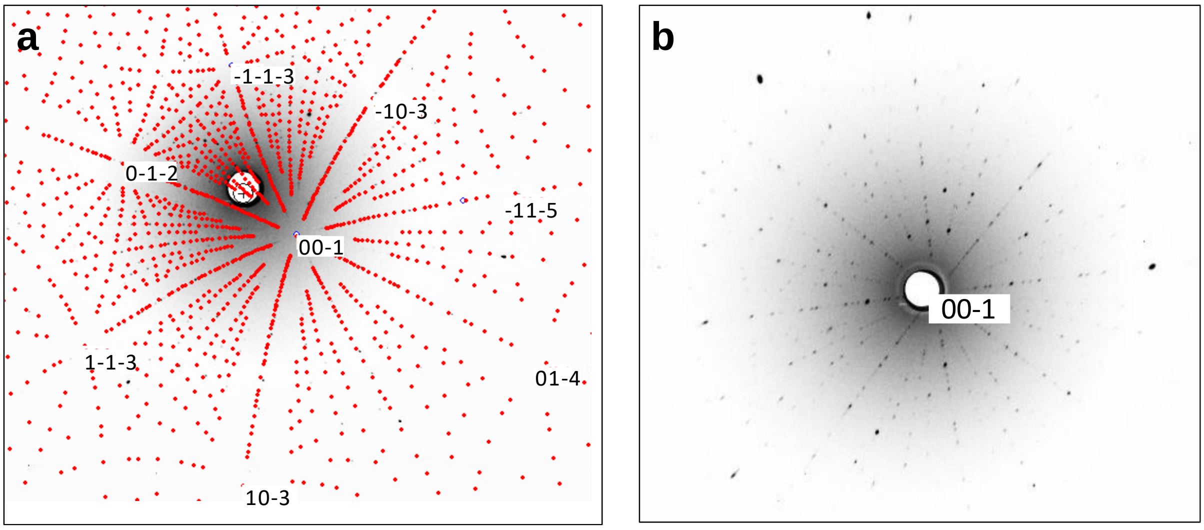

Figure S6. Laue diffraction pattern for MnAlGe crystal (a) before and (b) after orientation along [001] direction respectively. The diffraction pattern of the crystal can be indexed based on the space group *P*4/*nmm*. The oriented crystal was cut into rectangular bars using a wire saw for transport and magnetization measurements.

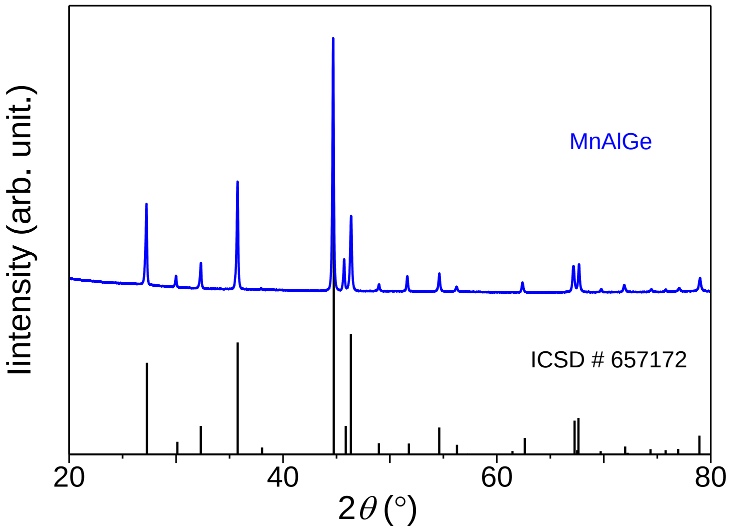

Figure S7. Powder X-ray diffraction (PXRD) pattern of the crushed MnAlGe crystal (λ = 1.54056 Å).

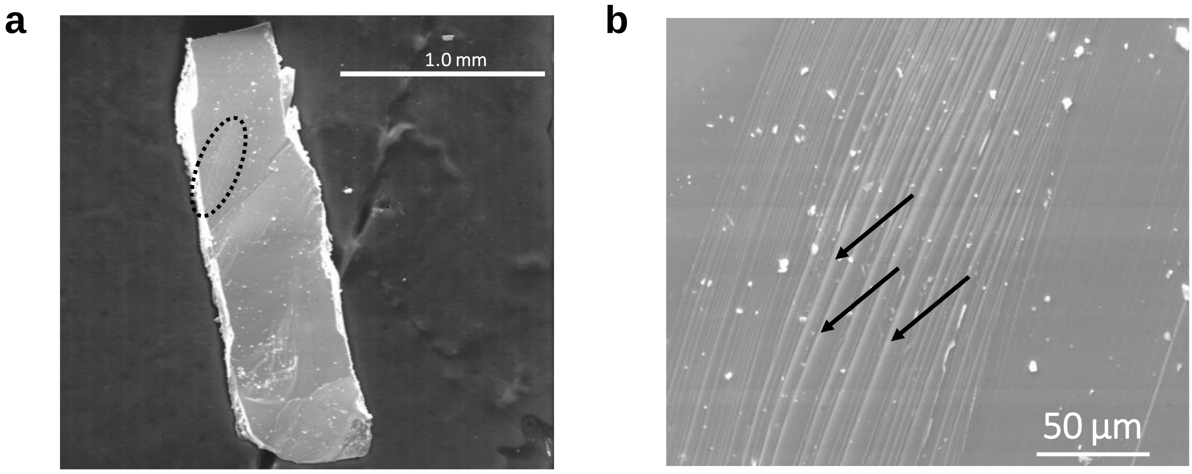

Figure S8. (a) SEM image of a small broken MnAlGe crystal. The dotted portion of (a) is magnified and shown in (b), indicating the layered nature of the crystal.

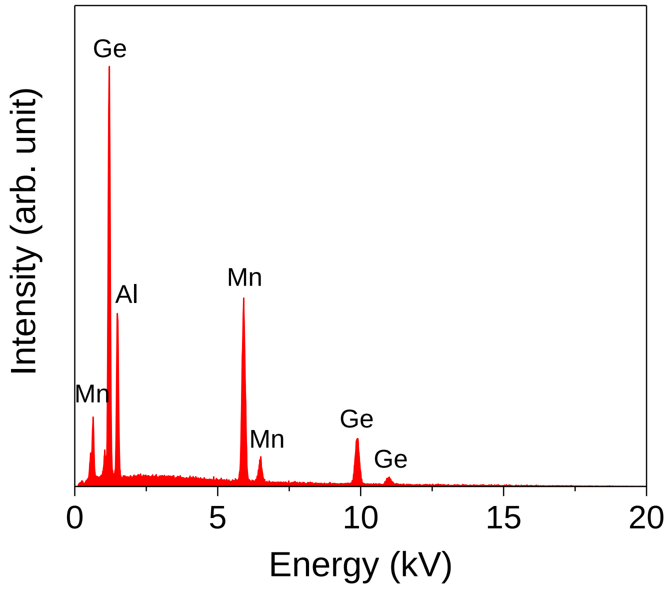

Figure S9. Energy Dispersive X-ray Spectroscopy (EDXS) spectra of MnAlGe

Table S1. Composition of the MnAlGe crystal determined by Energy Dispersive X-ray Spectroscopy (EDXS) and Inductively coupled plasma optical emission spectroscopy (ICP-OES).

Element	ICP composition (Wt %)	EDXS composition (Wt %)
Mn	36.28	35.69
Al	17.17	16.86
Ge	46.33	47.45

The calculated average atomic ratio from the ICP-OES is Mn : Al : Ge = 1.03 : 1: 1
The EDXS average composition is Mn : Al : Ge = 1 : 0.9619 : 1.005 . The Al deficiency in EDXS composition might be an artifact related to small atomic mass of Aluminum since the detection errors EDXS are generally high for lighter elements.

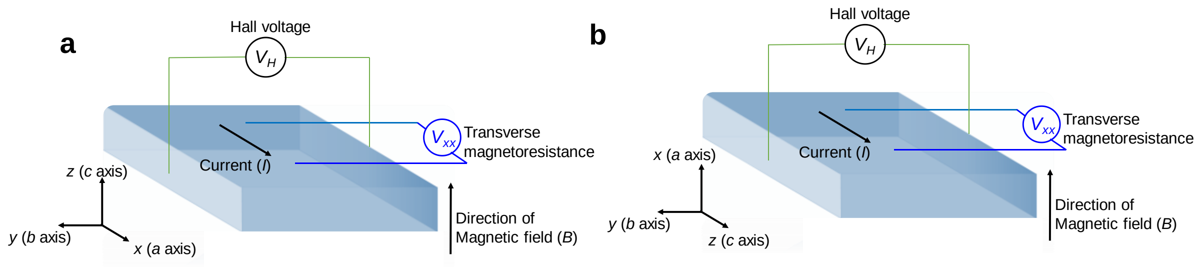

Figure S10. Schematic geometry for the electrical measurements. (a) Current (*I*) was applied within the *ab*-plane. The magnetic field (*B*) was applied parallel to the *c*-axis. (b) Current (*I*) was applied in the *c*-axis. The magnetic field (*B*) was applied parallel to the *ab*-plane.

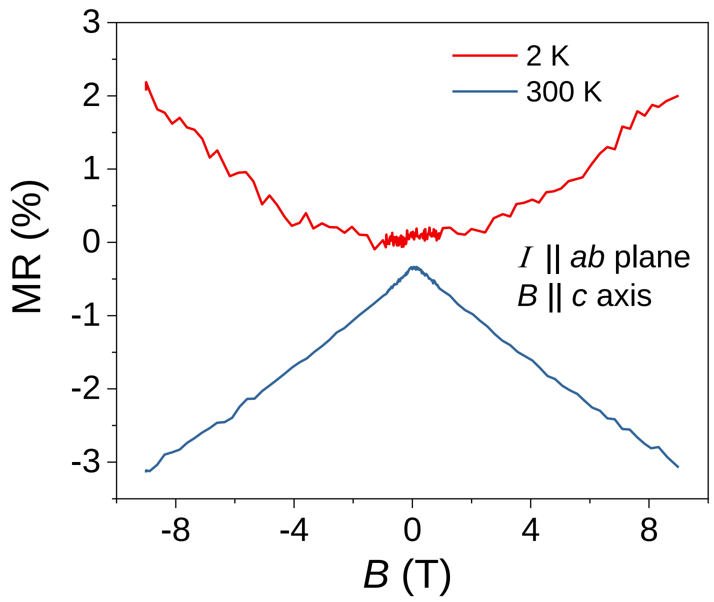

Figure S11. Magnetic field dependent transverse magnetoresistance (MR) for MnAlGe at selected temperatures.

Compared to high mobility non-magnetic topological semimetal, magnetic topological semimetals exhibit a lower magnetoresistance (MR). Such an observation is related to the lower carrier mobility of the material. The low carrier mobility (μ) ~46.62 $cm^2V^{-1}s^{-1}$ (see discussion on carrier concentration and mobility) results in a small MR for MnAlGe. Many recently discovered topological magnets such as Co_2MnGa, Fe_3GeTe_2, Fe_3Sn_2, $Co_3Sn_2S_2$ also exhibit small MR values due to low carrier mobilities.[1-4]

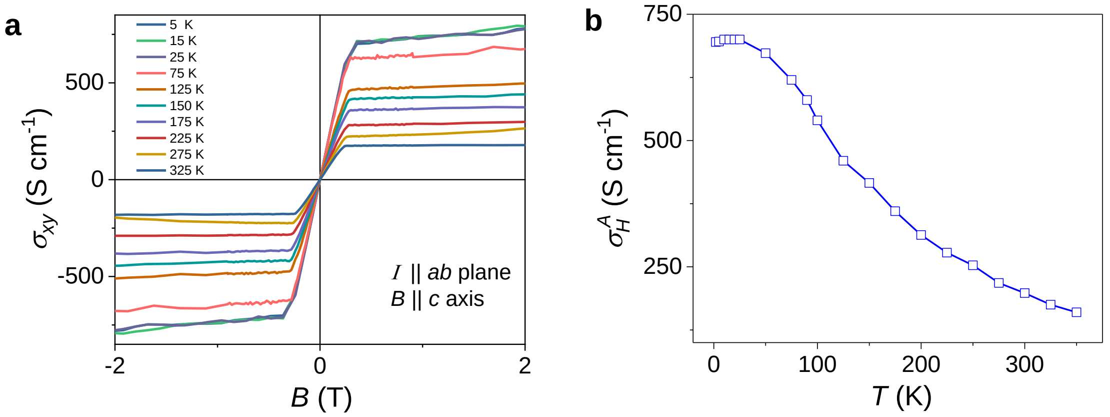

Figure S12. Additional transport data of a MnAlGe crystal. (a) Magnetic field dependence of the Hall conductivity (σ_{xy}) and (b) temperature dependence of Hall conductivity (σ_{xy}).

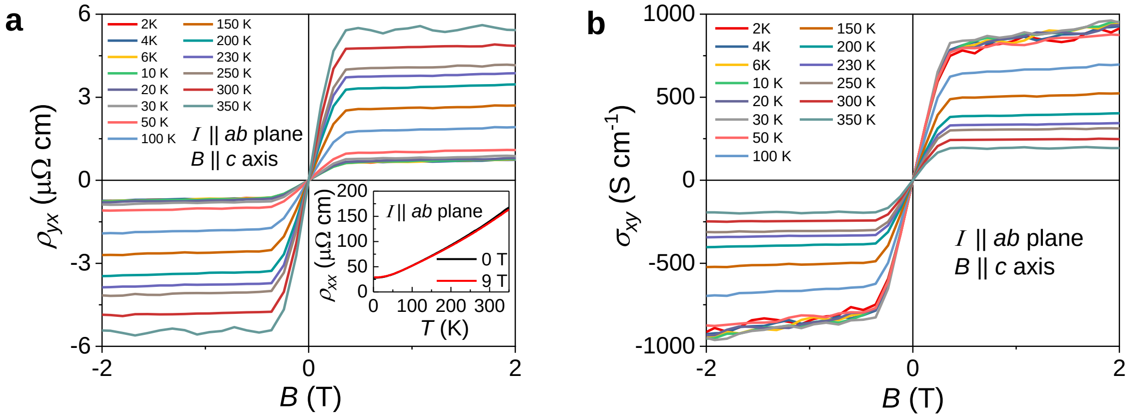

Figure S13. Transport data on a second MnAlGe crystal. (a) Magnetic field dependence of the Hall resistivity (ρ_{yx}) and resistivity (ρ_{xx}, inset) in fields of 0 and 9 T at selected temperatures. (b) Hall conductivity (σ_{xy}).

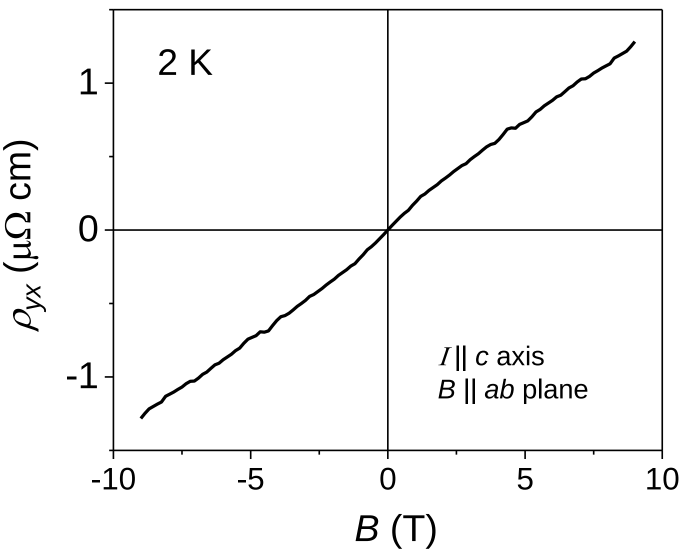

Figure S14. Magnetic field dependence of the Hall resistivity (ρ_{yx}), where electrical current applied along the *c*- axis and magnetic field (*B*) applied within the *ab*- plane. The linear behavior of the curve indicates no anomalous transport.

Carrier concentration and mobility for MnAlGe.

The Hall coefficient (R_H) was extracted with a linear fit to the ρ_{yx}-B data of Figure S14. The carrier concentration (n) was calculated using $n = 1/R_He$ where, e is the elementary charge, and the mobility (μ) using $\mu = R_H/\rho_{xx}$ where, ρ_{xx} is resistivity.
The estimated carrier concentration (n) is 4.287×10^{21} cm^{-3} at 2 K.
The c-axis cell parameter for MnAlGe is 5.933 Å, which is the distance between the neighboring magnetic Mn layers. Therefore, the carrier density per magnetic layer sheet is
5.933×10^{-8} cm $\times$ 4.287×10^{21} cm^{-3}
$= 2.54 \times 10^{14}$ cm^{-2}
The estimated mobility at 2 K is 46.62 cm^2 V^{-1} s^{-1}.

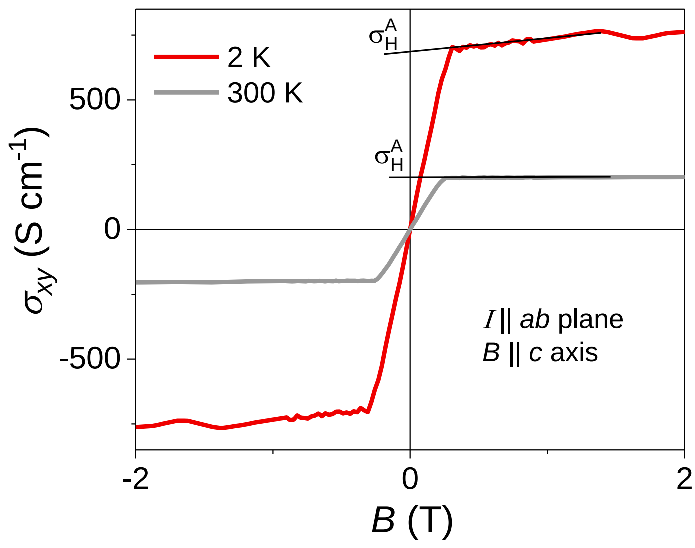

Figure S15. Plot showing an estimate of the anomalous Hall conductivity (σ_H^A). The anomalous response has been estimated by extrapolating the slope to the zero magnetic field.

Angular-dependent measurements of the anomalous Hall effect. To understand the large anisotropic out-of-plane magnetization and 2D nature of Berry curvature in the compound we have carried out angular-dependent measurements of the AHE at various current configurations at 2 K (Figure S16a). The current (I) was applied along a-axis. The magnetic field (B) was applied parallel to the c-axis. The Hall voltage was measured perpendicular to the applied current in the ab-plane. In case of $\theta = 0°$, the magnetic field was applied along the c-axis, which was orthogonal to both applied current and Hall voltage leads. For angle dependent measurement, the magnetic field was rotated toward the in-plane direction along the applied current. Therefore, in case of measurement at $\theta = 90°$ the magnetic field is directly along the direction (a-axis) of the applied current. The AHE ($\rho_{yx}^A, \sigma_{xy}^A$) remains almost constant with increasing θ and suddenly changes sign at θ = 90° (Figure S16b,c). A similar change of sign happens again at $\theta = 270°$. Although the strength of the applied magnetic field along the c-axis decreases with increasing θ, a component of the field maintains the out-of-plane magnetization below $\theta = 90°$. At angles greater than 90°, the magnetic field changes its direction, resulting in

a change in the direction of the spin, which results in the sign change in the AHE. A similar phenomenon occurs at 270°. We have also measured magnetic field dependence of ρ_{yx} at different angular configuration (Figure S16d). With increase of angle (θ), the slope of the ρ_{yx} vs. B changes while ρ_{yx}^{A} remains almost constant and suddenly changes sign at θ = 90°. This observation supports the large anisotropic out-of-plane magnetization in the compound. Moreover, a component of the field (at θ ≠ 90) always lies along the *c*-axis that results in an out-of-plane magnetization which is consistent with the 2D-type cylindrical Berry curvature for the *z*-component.

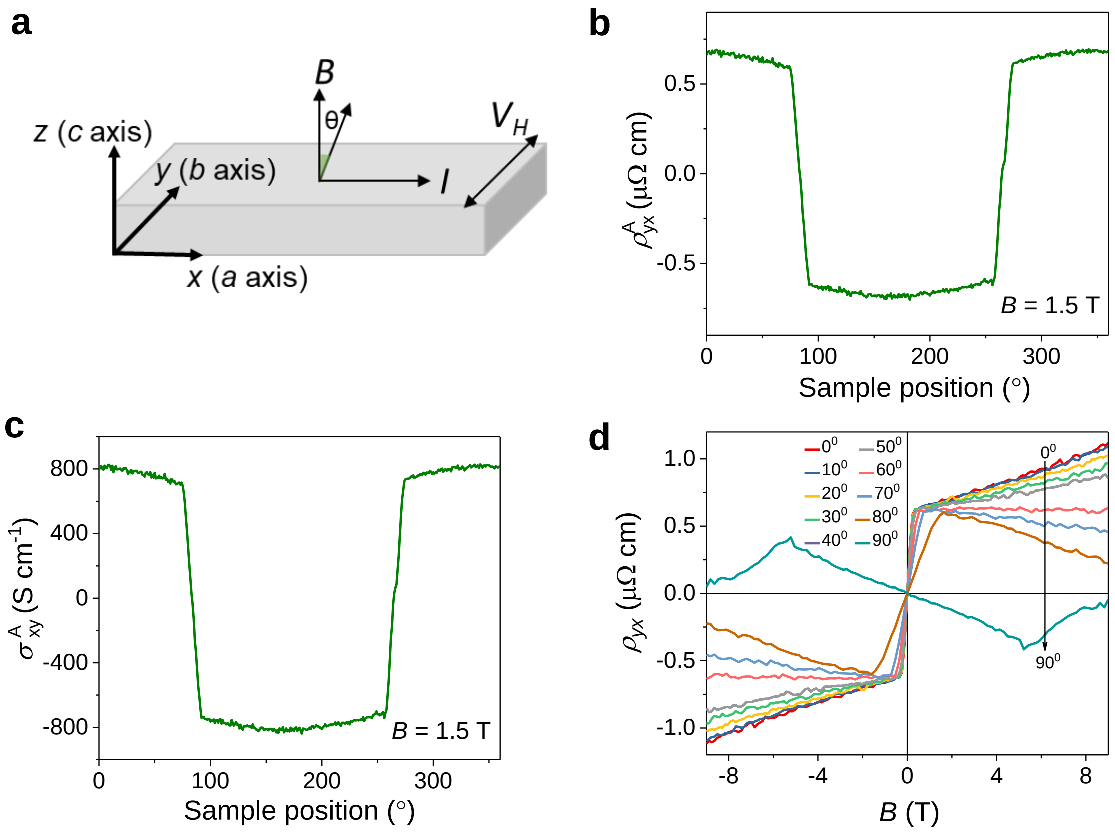

Figure S16. (a) Schematic of the sample geometry used for angle (θ) dependent AHE measurement. Angular (θ) dependence of the (b) anomalous Hall resistivity (ρ_{yx}^{A}) and (c) anomalous Hall resistivity (σ_{xy}^{A}). (d) Field dependence of Hall resistivity (ρ_{yx}) at different angular configuration.

Magnetization (M) dependence of anomalous Nernst thermopower (S_{xy}^{A}). The anomalous Nernst effect (ANE) for topologically trivial ferromagnets is proportional to the magnetization of the material. The linear scaling relation for conventional ferromagnetic metals can be express as, $|S_{xy}^{A}| = |Q_{xy}^{A}|\mu_0 M$, where, S_{xy}^{A} is anomalous Nernst thermopower, $\mu_0 M$ is magnetization in Tesla unit and Q_{xy}^{A} is anomalous Nernst coefficient. The Q_{xy}^{A} for trivial ferromagnets ranges from ~0.05 μV K^{-1} T^{-1} to ~1 μV K^{-1} T^{-1}. Considering the scaling relation, a maximum S_{xy}^{A} expected for MnAlGe is ~0.027 μV K^{-1} to ~0.5403 μV K^{-1}, which is lower than measured value. Therefore, S_{xy}^{A} for MnAlGe does not follow the conventional scaling relation and indicating a topological band structural effect as the origin. A similar observation is reported for Mn_3Sn, Co_2MnGa, $Co_3Sn_2S_2$.[5-8]

Intrinsic vs extrinsic anomalous Hall effect. In order to understand the origin of AHE in MnAlGe, we fit the experimental anomalous hall resistivity data with the longitudinal Hall resistivity using the equation S1:

$$\rho_{yx}^{A}(T) = (\alpha\rho_{xx0} + \beta\rho_{xx0}^{2}) + \gamma\rho_{xx}^{2}(T) \qquad \text{(S1)}$$

where ρ_{xx0} is residual longitudinal resistivity. The first term (α) is from the contribution of skew scattering while the second term (β) contains the contribution of side-jump and γ is intrinsic Berry curvature contribution.[10,11] In Figure S17 we present the plot of ρ_{yx}^{A} versus ρ_{xx}^{2}. Now we would like to point out that the nonlinear behaviour of the fittings. In the above empirical relations, there are extrinsic term, which depends on residual resistivity/conductivity (independent of temperature) and intrinsic term (dependent on temperature). In other words, former one belongs to elastic and later one is to inelastic scatterings.[13] Shitade *et al.* also pointed out that the role of above scattering processes are different and temperature dependence

of intrinsic contribution has been identified in Ni thin films.[11,13] Such a deviation can be seen in many other compounds $Co_3Sn_2S_2$, Fe_3GeTe_2, Fe_3Sn_2 but its origin is not very much clear.[2,4,12] Among them, the shifting of chemical potential with temperature through small SOC gap is the most common and plausible, where AHE is typically constants only through the SOC gap.[14] On the other hand, the effect of magnetization are responsible for nonlinear behavior in Mn_5Ge_3.[15,16]

In the present compound, the magnetization changes only 5% from 2-300K and therefore magnetization is not a prominent factor. However, similar to other materials like Ni thin films change of chemical potential through SOC gap is most promising factor in MnAlGe since AHC presents a sharp peak near E_F (Figure 2f). In particular since the topological states (band anti-crossings) in the topological materials are mostly concentrated near the Fermi energy, a small shift in E_F will have large change in AHC compared to materials like Ni, Fe etc. where the Berry curvature is more or less equally distributed in the whole occupied bands. Since topological states (nodal line) in MnAlGe is quite close to E_F, a large change in AHC is expected with change in temperature, leading to a nonlinear behavior in the scaling law. Therefore a rough linear fitting of the ρ_{yx}^{A} vs ρ_{xx}^{2} at low temperature region gives an intrinsic AHC of ≈ 560 S/cm.

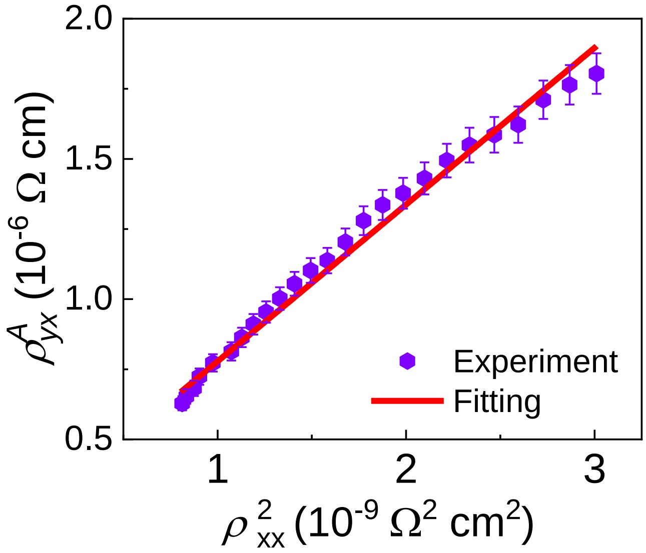

Figure S17. Scaling relation between the anomalous Hall resistivity (ρ^A_{yx}) to resistivity (ρ_{xx}) for MnAlGe. Error bars in the plot specify uncertainty in the measurements.

Table S2. Distance between magnetic layers, ferromagnetic transition temperature and Anomalous Hall conductivity (AHC) of different layered materials.

Compound	Distance between magnetic layers (Å)	Transition temperature, T_c (K)	|AHC| (S cm^{-1}) @2 K	Reference
$Fe_{3-x}GeTe_2$	8.16	220	540	[2]
Fe_3Sn_2	6.60	670	1100	[3]
$Co_3Sn_2S_2$	4.39	177	1130	[4]
$Fe_{1/4}TaS_2$	6.07	160	336	[9]
MnAlGe	5.93	503	700	Present work

Reference

[1] A. Sakai, Y. P. Mizuta, A. A. Nugroho, R. Sihombing, T. Koretsune, M. T. Suzuki, N. Takemori, R. Ishii, D. Nishio-Hamane, R. Arita, P. Goswami, S. Nakatsuji, *Nat. Phys.* 2018, **14**, 1119.

[2] K. Kim, J. Seo, E. Lee, K. T. Ko, B. S. Kim, B. G. Jang, J. M. Ok, J. Lee, Y. J. Jo, W. Kang, J. H. Shim, C. Kim, H. W. Yeom, B. Il Min, B.-J. Yang, J. S. Kim, *Nat. Mater.* **2018**, 17, 794.

[3] L. Ye, M. Kang, J. Liu, F. von Cube, C. R. Wicker, T. Suzuki, C. Jozwiak, A. Bostwick, E. Rotenberg, D. C. Bell, L. Fu, R. Comin, J. G. Checkelsky, *Nature* ***2018***, 555, 638.

[4] E. Liu, Y. Sun, N. Kumar, L. Muechler, A. Sun, L. Jiao, S.-Y. Yang, D. Liu, A. Liang, Q. Xu, J. Kroder, V. Süß, H. Borrmann, C. Shekhar, Z. Wang, C. Xi, W. Wang, W. Schnelle, S. Wirth, Y. Chen, S. T. B. Goennenwein, C. Felser, *Nat. Phys.* **2018**, 14, 1125.

[5] M. Ikhlas, T. Tomita, T. Koretsune, M. T. Suzuki, D. Nishio-Hamane, R. Arita, Y. Otani, S. Nakatsuji, *Nat. Phys.* **2017**, *13*, 1085.

[6] X. Li, L. Xu, L. Ding, J. Wang, M. Shen, X. Lu, Z. Zhu, K. Behnia, *Phys. Rev. Lett.* 2017, **119**, 056601.

[7] S. N. Guin, K. Manna, J. Noky, S. J. Watzman, C. Fu, N. Kumar, W. Schnelle, C. Shekhar, Y. Sun, J. Gooth, C. Felser, *NPG Asia Mater.* **2019**, *11*, 16.

[8] S. N. Guin, P. Vir, Y. Zhang, N. Kumar, S. J. Watzman, C. Fu, E. Liu, K. Manna, W. Schnelle, J. Gooth, C. Shekhar, Y. Sun, C. Felser, *Adv. Mater.* **2019**, *31*, 1806622.

[9] J. G. Checkelsky, M. Lee, E. Morosan, R. J. Cava, N. P. Ong, *Phys. Rev. B* **2008**, *77*, 014433.

[10] Y. Tian, L. Ye, X. Jin, *Phys. Rev. Lett.* **2009**, *103*, 087206.

[11] L. Ye, Y. Tian, X. Jin, D. Xiao, *Phys. Rev. B* **2012**, *85*, 220403(R).

[12] I. Belopolski, K. Manna, D. S. Sanchez, G. Chang, B. Ernst, J. Yin, S. S. Zhang, T. Cochran, N. Shumiya, H. Zheng, B. Singh, G. Bian, D. Multer, M. Litskevich, X. Zhou, S.-M. Huang, B. Wang, T.-R. Chang, S.-Y. Xu, A. Bansil, C. Felser, H. Lin, M. Z. Hasan, *Science* **2019**, *1281*, 1278.

[13] A. Shitade, N. Nagaosa, *J. Phys. Soc. Jpn.* **2012**, *81*, 083704.

[14] D. Xiao, M. -C. Chang, Q. Niu, *Rev. Mod. Phys.* **2010**, *82*, 1959.

[15] C. Zeng, Y. Yao, Q. Niu, H.H. Weitering, *Phys. Rev. Lett.*, **2006**, *96*, 037204.

[16] R. Mathieu, A. Asamitsu, H. Yamada, K. S. Takahashi, M. Kawasaki, Z. Fang, N. Nagaosa, and Y. Tokura, *Phys. Rev. Lett.*, **2004**, *93*, 016602.
